# Multiple-Color Reflectors Using Bichiral Liquid Crystal Polymer Films and Their Applications in Liquid Crystal Displays

**DOI:** 10.3390/polym12123031

**Published:** 2020-12-17

**Authors:** Cheng-Kai Liu, Ming-Hsien Li, Chi-Lun Ting, Andy Ying-Guey Fuh, Ko-Ting Cheng

**Affiliations:** 1 Department of Optics and Photonics, National Central University, Taoyuan 320, Taiwan; aerobert007@gmail.com; 2 Department of Applied Materials and Optoelectronic Engineering, National Chi Nan University, Nantou County 545, Taiwan; mhli1125@ncnu.edu.tw; 3 Department of Photonics, National Cheng Kung University, Tainan 701, Taiwan; chaser1213@yahoo.com.tw; 4 Department of Physics, National Cheng Kung University, Tainan 701, Taiwan

**Keywords:** cholesteric liquid crystals, multiple-color reflectors, chiral polymers, liquid crystal polymers, color gamut, light-utilization efficiency

## Abstract

Multiple-color reflectors using bichiral liquid crystal polymer films (BLCPFs) are investigated. The BLCPFs consist of alternate layers of two different single-pitch cholesteric liquid crystal (CLC) layers, named CLC#A and CLC#B. The thickness of each CLC layer equals its single pitch length. The optical properties in terms of reflections, reflection-wavelength ranges, and distributions of reflection spectra of the BLCPFs that result from the fixed pitch length of CLC#A along with the decrease of the pitch length of CLC#B are qualitatively simulated and investigated. The results indicate that the above optical properties of the BLCPFs depend on the LC birefringence and pitch lengths of CLC#A and CLC#B layers. The concept of fabrication method of the BLCPFs by using polymerizable CLCs and thin films of poly(vinylalcohol) or photoalignment materials is discussed. They have potential practical applications in functional color filters, asymmetrical transmission systems, etc., owing to the multiple reflection bands of BLCPFs. Moreover, the BLCPFs, which can enhance the color gamut and light-utilization efficiency of light sources/LC displays, are reported herein.

## 1. Introduction

Manipulations of wavelength and reflection of reflection light can be realized while using one-dimensional photonic crystals (1D-PCs) that consist of alternate layers of two dielectric materials. The reflection and reflection-wavelength range of 1D-PCs depend on the thicknesses of the two dielectric materials and the refractive index difference between them [1]. Common ways of fabricating 1D-PCs that are based on layer-by-layer depositions have been reported [2,3,4].

Cholesteric liquid crystals (CLCs), which are also called chiral nematic LCs (NLCs), can be considered to be 1D-PCs, owing to their periodic twist structures [5,6]. CLCs with a single pitch usually possess a single reflection band, and the reflection-wavelength range depends on CLC pitch length, the ordinary and extraordinary refractive indexes (n_o_ and n_e_) of the LCs, and others. Aiming to obtain multiple reflection bands while using single-pitch CLCs, Ha et al. successfully demonstrated a reflector comprising alternate layers of polymer single-pitch CLCs and isotropic layers in order to realize simultaneous red-green-blue reflection colors [7]. Gao et al. and Gevorgyan et al. studied reflectors comprising alternative layers of CLCs and an isotropic material [8,9,10]. Ha et al. further investigated bichiral LC films comprising two CLC layers with an isotropic PVA film in between as a defect layer and found that the optical properties of the bichiral LC films are polarization independent. They also demonstrated the color-temperature tunability of a reflector while using bichiral LC films [11,12]. The optical properties of single-pitch CLC films, which possess quasi-periodic Fibonaccian defects, have also been investigated [13]. Sabah et al. also reported optical properties of a chiral mirror comprising alternating chiral layers [14].

Bichiral structures (so-called chiral periodic/periodic chiral structures) can also be realized while using chiral metamaterials. These bichiral structures can be applied to microwave filters or antenna systems [15]. The optical properties of periodic chiral structures (periodic chiral media) and their applications as polarization-/wavelength-dependent filters, devices of distributed-feedback, etc., have been reported [16,17,18,19]. Moreover, light scattering that results from a grating based on bichiral structures (chiral periodic structures) and applications of bichiral structures for optical fibers have been explored [20,21,22].

In the present study, multiple-color bichiral LC polymer films (BLCPFs) are investigated. The BLCPFs consist of alternate layers of two different single-pitch CLC layers, namely, CLC#A and CLC#B. The thicknesses of a single layer of CLC#A (CLC#B) equal a single pitch length of CLC#A (CLC#B). The complete changes in reflections, reflection-wavelength ranges, and distributions of reflection spectra of the BLCPFs, which result from the fixed pitch length of CLC#A and the decrease in pitch length of CLC#B, are qualitatively investigated. The LC birefringence and the pitch lengths of CLC#A and CLC#B layers play important roles in the optical properties of reflection spectra of the BLCPFs. We also report a conceptual method of fabricating the BLCPFs using a thin film of poly(vinylalcohol) (PVA) or photoalignment materials and CLC polymers. The BLCPFs have potential for applications as functional color filters (CFs), asymmetrical transmission systems, etc. Furthermore, the BLCPFs, which simultaneously enhance the light-utilization efficiency and color gamut of light sources/LC displays, are reported herein.

## 2. Simulation Method

**Figure 1:**
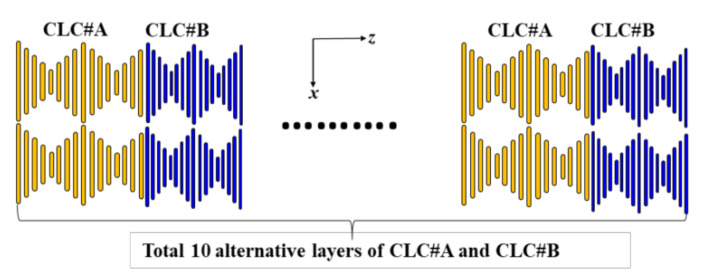
Schematics of using bichiral liquid crystal polymer films (BLCPFs) comprising alternate layers of cholesteric liquid crystal#A (CLC#A) and CLC#B.

The simulation method used herein is based on the Berreman 4 × 4 matrix while using commercial 1D-DIMOS software (Display-Messtechnik & Systeme GmbH & Co., KG, Rottenburg am Neckar, Germany) [23]. Figure 1 presents the schematics of the BLCPFs comprising alternate layers of CLC#A and CLC#B, where the substrates are not considered in the simulation. The thickness of a single layer of CLC#A (CLC#B) equals a single pitch length of CLC#A (CLC#B). The BLCPFs consist of 10 cycles of alternate layers of CLC#A and CLC#B. The directions of chiral handedness of CLC#A and CLC#B are identical. An unpolarized broadband light beam, whose wavelength ranges from 400 nm to 1500 nm, is used as a light source for simulation. For simplicity, the refractive-index dispersion of the LCs that were used in the present simulations is not considered [24,25]. Helical axes of all CLC layers are uniform and along the ***z***-axis; light scattering is not considered in all simulations. All of the incident lights travel along the ***z***-axis.

## 3. Results and Discussions

### 3.1. Changes in Reflection Spectra of BLCPFs by Adjusting the Pitch Lengths and Refractive Indexes of LCs

**Figure 2:**
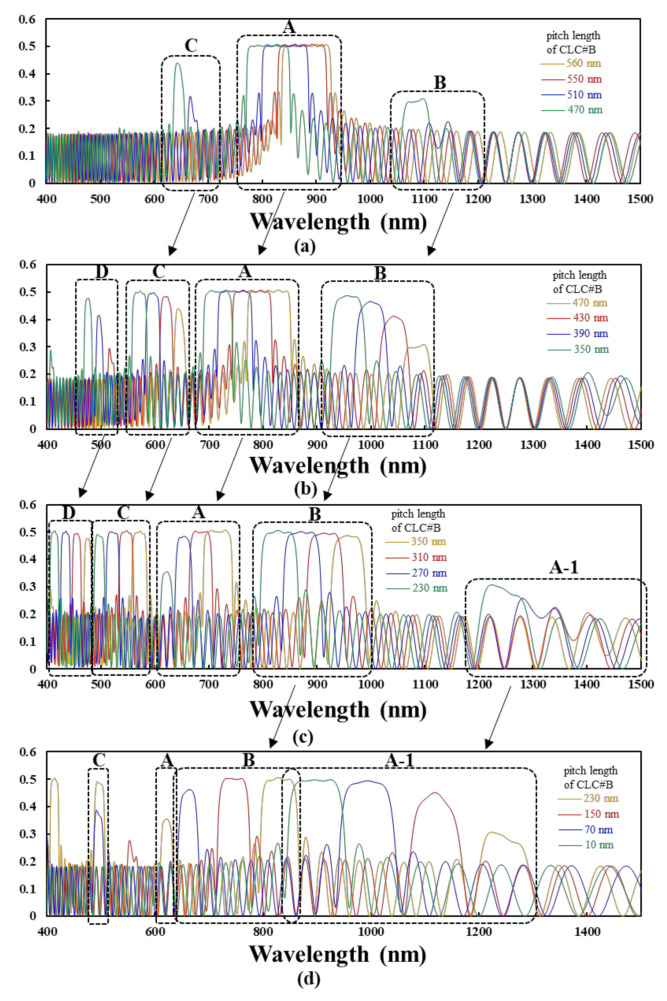
Simulation results of the reflection spectra of BLCPFs realized by fixing the pitch length of CLC#A at 560 nm and by decreasing the pitch length of CLC#B from (**a**) 560 [yellow curve], to 550 [red curve], 510 [blue curve], and 470 [green curve] nm; (**b**) 470 [yellow curve], to 430 [red curve], 390 [blue curve], and 350 [green curve] nm; (**c**) 350 [yellow curve], to 310 [red curve], 270 [blue curve], and 230 [green curve] nm; and, (**d**) 230 [yellow curve], to 150 [red curve], 70 [blue curve], and 10 [green curve] nm.

The complete changes in all reflection spectra that are induced by fixing the pitch length of CLC#A at 560 nm and by decreasing the pitch length of CLC#B from 560 nm to 10 nm are qualitatively demonstrated. Figure 2 presents the simulation results of the reflection spectra. The n_o_ (n_e_) of the LCs set herein for the CLC#A and CLC#B layers is 1.5 (1.65). Some reflection bands located on the wavelength below 400 nm can be obtained when the pitch length of CLC#B decreases, and we focus on the changes in reflection spectra with wavelength ranging from 400 nm to 1500 nm. We initially describe the complete changes in the reflection spectra of BLCPFs and then summarize the rules in designing the reflection spectra of BLCPFs for practical applications. The curves presenting the cases with the pitch length of CLC#B of 470 nm [350 nm] {230 nm} that are plotted in Figure 2a,b [Figure 2b,c] {Figure 2c,d} are the same.

Figure 2a–c present that the full width at half maximum (FWHM), the central reflection wavelength (CRW), and the reflection of CRW of reflection band (circled by a black dashed frame A) shrinks, blue-shifts, and keeps exceeding 0.48 by decreasing the pitch length of CLC#B from 560 to 550, 510, 470, 430, 390, 350, 310, and 270 nm, respectively. Table 1a shows the simulation results. The reflection of CRW starts decreasing to below 0.48 when the pitch length of CLC#B decreases to 230 nm. The reflection disappears when the pitch length of CLC#B decreases to 150 nm.

Figure 2a–c show that the reflection of CRW of reflection band (circled by a black dashed frame B) becomes greater than 0.25 when the pitch length of CLC#B decreases to 470 nm; it increases with decreasing the pitch length of CLC#B from 470 to 430, and 390 nm. The reflection of CRW becomes greater than 0.48 when the pitch length of CLC#B decreases to 350 nm. Table 1b shows the variations of FWHM, CRW, and reflection of CRW of reflection band (circled by a black dashed frame B) when the pitch length of CLC#B are 350, 310, 270, 230, and 150 nm. All of the reflections of CRW that are shown in Table 1b are greater than 0.48. The reflection of CRW decreases to below 0.48 when the pitch length of CLC#B decreases to 70 nm. The reflection band disappears when the pitch length of CLC#B further decreases to 10 nm.

Figure 2a–c present that the reflection of CRW of reflection band (circled by a black dashed frame C) becomes greater than 0.25 when the pitch length of CLC#B decreases to 510 nm; it increases with decreasing the pitch length of CLC#B from 510 nm to 470 nm. The reflection of CRW becomes greater than 0.48 when the pitch length of CLC#B decreases to 430 nm. The variations of FWHM, CRW, and reflection of CRW of reflection band (circled by a black dashed frame C) are shown in Table 1c when the pitch length of CLC#B are 430, 390, 350, 310, 270, and 230 nm. All of the reflections of CRW that are shown in Table 1c are greater than 0.48. The reflection band disappears when the pitch length of CLC#B decreases to 150 nm.

Figure 2b,c indicate that the reflection of CRW of reflection band (circled by a black dashed frame D) becomes greater than 0.25 when the pitch length of CLC#B decreases to 430 nm; it increases with decreasing the pitch length of CLC#B from 430 nm to 350 nm. The reflection of CRW becomes greater than 0.48 when the pitch length of CLC#B decreases to 310 nm. The variations of FWHM, CRW, and reflection of CRW of reflection band (circled by a black dashed frame D) are shown in Table 1d when the pitch lengths of CLC#B are 310, 270, and 230 nm. All of the reflections of CRW that are shown in Table 1d are greater than 0.48. The wavelength range of the reflection band is below 400 nm when the pitch length of CLC#B decreases to 150 nm.

Figure 2c,d show that the reflection of CRW of reflection band (circled by a black dashed frame A-1) becomes greater than 0.25 when the pitch length of CLC#B decreases to 230 nm; it increases with a decrease of the pitch length of CLC#B from 230 nm to 10 nm. The reflection of CRW becomes greater than 0.48 when the pitch length of CLC#B decreases to 70 nm.

Table 1a–d summarize the values of the FWHM and CRW, as well as the reflection of CRW of the reflection bands with reflections that are close to 0.5 (>0.48) in the black dashed frames A–D plotted in Figure 2, respectively. The reflection of CRW is rounded to the second decimal place. All of the reflections blue-shift with the decrease of the pitch length of CLC#B. The FWHM presented in Table 1a decreases with the decrease of the pitch length of CLC#B, whereas the FWHM in Table 1b,c,d initially increases and then decreases when the pitch length of CLC#B decreases.

**Figure A1:**
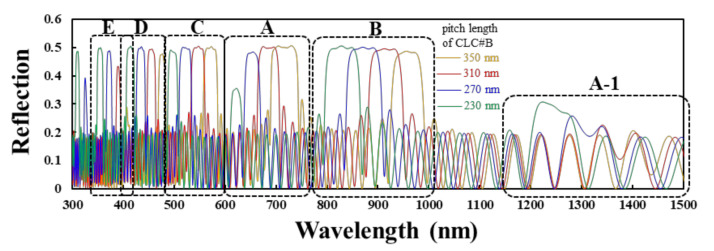
Simulation results of the reflection spectra of BLCPFs, with wavelength ranging from 300 nm to 1500 nm, realized by fixing the pitch length of CLC#A at 560 nm and by decreasing the pitch length of CLC#B from 350, to 310, 270, and 230 nm.

The rules for designing the reflection spectra of BLCPFs for real applications are summarized based on the simulation results that are shown in Figure 2 and Table 1. The reflection of the blue reflection band in the black dashed frame A in Figure 2c starts decreasing when the pitch length of CLC#B decreases to 270 nm from 560 nm; it decreases to below 0.4 when the pitch length of CLC#B decreases to 230 nm. Moreover, the reflection of each reflection band in the black dashed frames A, B, C, and D in Figure 2c reaches around 0.5 when the pitch length of CLC#B decreases to 310 nm from 560 nm. Table 1a–d also show that all of the reflections of CRW are close to 0.5 when the pitch length of CLC#B is around 310 nm. Accordingly, the first rule is that the pitch length of CLC#B should be around half of the pitch length of CLC#A in order to ensure that all reflections of reflection bands in the black dashed frames A, B, C, and D in Figure 2b,c are close to 0.5. In the in Appendix A, Figure A1 shows the results plotted in Figure 2c with the wavelength range from 300 nm to 1500 nm. The reflection band in the black dashed frame E appears when the pitch length of CLC#B decreases to 350 nm; its reflection becomes greater than 0.43 when the pitch length of CLC#B decreases to around half of the pitch length of CLC#A. The reflection band can be applied to LC displays if it is located within the visible light range by increasing the pitch length of CLC#A. The second rule is that the reflection bands red-shift (blue-shift) when the pitch length of CLC#A is fixed and that of CLC#B increases (decreases). The second rule is similar to that of 1D-PCs. The reflections and bandwidths of the reflection bands presented in Figure 2 can reach 0.5 and expand if the birefringence of the LCs could be further increased, respectively. This finding is discussed in the next paragraph.

**Figure 3:**
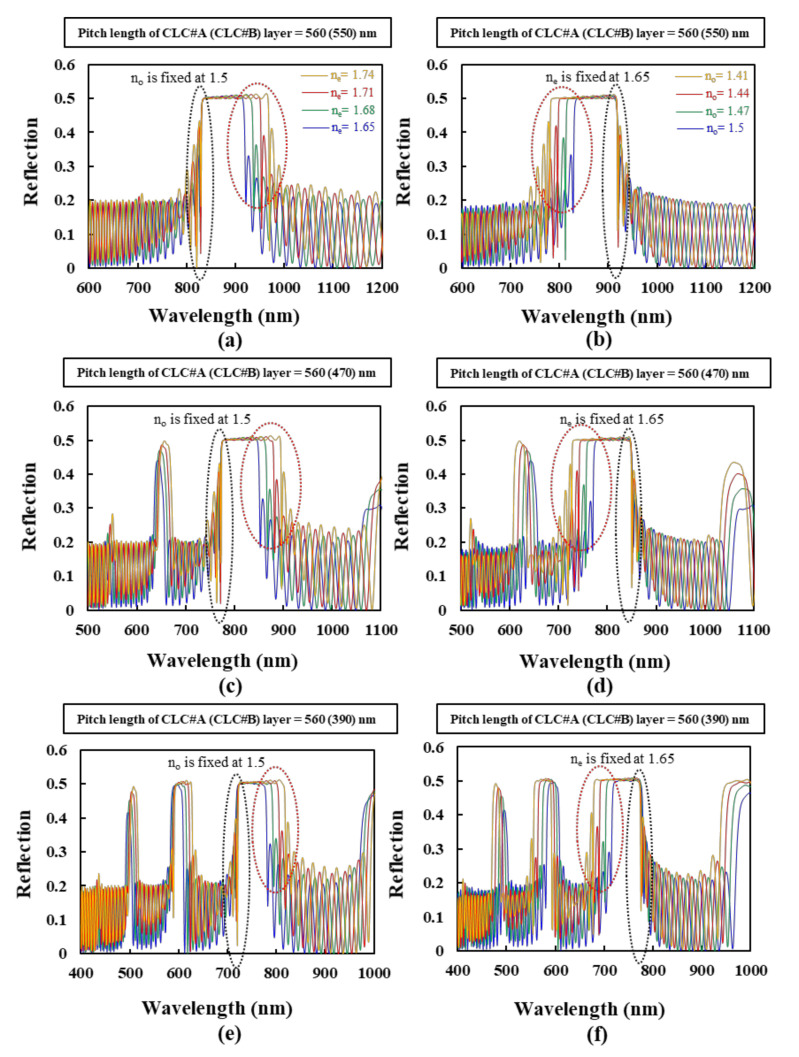
The simulation results of the reflection spectra of BLCPFs when the pitch length of CLC#A is fixed at 560 nm, and the pitch lengths of CLC#B are (**a**,**b**) 550 nm, (**c**,**d**) 470 nm, and (**e**,**f**) 390 nm. The various n_e_ [n_o_] values of CLC#A and CLC#B of the blue, green, red, and yellow reflection spectra presented in (**a**,**c**,**e**) [(**b**,**d**,**f)**] are 1.65, 1.68, 1.71, and 1.74 [1.5, 1.47, 1.44, and 1.41], respectively; the fixed n_o_ (n_e_) of the LCs in (**a**,**c**,**e**) [(**b**,**d**,**f**)] is 1.5 [1.65].

**Figure 4:**
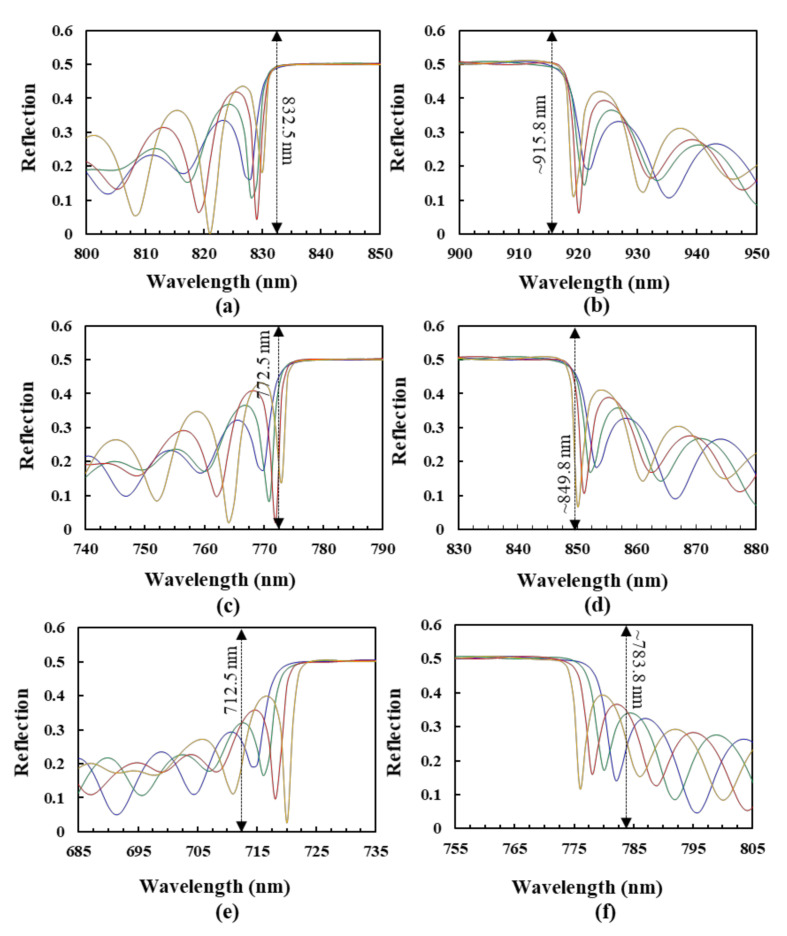
Simulation results of the reflection spectra of BLCPFs. (**a**–**f**) are the detailed reflection spectra in the black dashed circles plotted in Figure 3a–f, respectively.

This paragraph discusses the reflection spectra of the BLCPFs comprising CLC#A and CLC#B with various n_e_ and n_o_ values of the LCs. The simulation results of three different BLCPFs, made by CLC#A with a fixed pitch length (560 nm) and CLC#B with various pitch lengths of 550, 470, and 390 nm, are shown in Figure 3a,b, Figure 3c,d, and Figure 3e,f, respectively. Regarding the variations in refractive index, the blue, green, red, and yellow curves that are presented in Figure 3a,c,e show the simulated reflection spectra of the cases with a fixed n_o_ of 1.5 and various n_e_ values of 1.65, 1.68, 1.71, and 1.74, respectively. The blue, green, red, and yellow curves in Figure 3b,d,f depict the simulated reflection spectra of BLCPFs with a fixed n_e_ of 1.65 and various n_o_ values of 1.5, 1.47, 1.44, and 1.41, respectively. The small n_o_ values of 1.41 and 1.44 are just used in order to theoretically analyze the wavelength shifts of the reflection bands that are shown in Figure 3 and Figure 4, respectively. Figure 4a–f show the detailed reflection spectra of the reflection spectra circled in the black dashed circles presented in Figure 3a–f, respectively.

**Figure 5:**
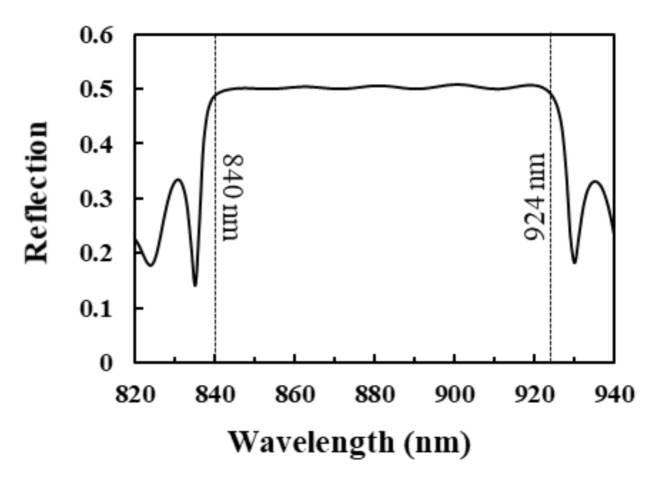
Simulated reflection spectrum of single-pitch CLCs with 20 turns of CLC helix, and the pitch length and the n_o_/n_e_ of the CLCs are 560 nm and 1.5/1.65, respectively.

The short [long] wavelength of the reflection-band edge in the black dashed circle presented in Figure 3a (Figure 4a) [Figure 3b (Figure 4b)] is almost invariant when the n_o_ [n_e_] of CLCs is fixed. The possible reason may be that the structures of the BLCPFs become more similar to those of single-pitch CLCs when the difference in the pitch length between CLC#A and CLC#B becomes as small as possible. The short [long] wavelength of the reflection-band edge of usual single-pitch CLCs is invariant when the n_o_ [n_e_] of the CLCs is fixed [5]. According to Figure 3a,c,e [Figure 3b,d,f], the amount of red-shift [blue-shift] of the long [short] wavelength of the reflection-band edge from the reflection spectrum which is marked by blue to that of the reflection spectrum which is marked by yellow (marked by the red dashed circles) is much larger than the amount of red-shift [blue-shift] of the short [long] wavelength of the reflection-band edge from the reflection spectrum which is marked by blue to that of the reflection spectrum which is marked by yellow (marked by the black dashed circles) when n_e_ [n_o_] increases [decrease] and n_o_ [n_e_] is fixed. Moreover, the amount of the red-shift [blue-shift] of the short [long] wavelength of the reflection-band edge from the reflection spectrum which is marked by blue to that of the reflection spectrum which is marked by yellow progressively enlarges with the increase of the difference in pitch length between CLC#A and CLC#B, as shown in Figure 4a,c,e [Figure 4b,d,f]. These results indicate that the effect of fixing n_e_/n_o_ of LCs in order to fix the long/short wavelength of the reflection-band edges of the BLCPFs progressively weakens when the difference in pitch length between CLC#A and CLC#B progressively enlarges. In order to further study the simulation results shown in Figure 3 and Figure 4, we find that the CRW of the reflection band (*λ*_cen_), the long wavelength of the reflection-band edge (*λ_long_*), and the short wavelength of the reflection-band edge (*λ_short_*) of each reflection band presented in Figure 4 can be roughly estimated while using the following equations [5,6,7],

(1) $$\lambda_{cen}=\frac{\left(n_{o}+n_{e}\right)}{2}\times \frac{\left(P_{CLC\#A}+P_{CLC\#B}\right)}{2}$$

(2) $$\lambda_{short}=n_{o}\times \frac{\left(P_{CLC\#A}+P_{CLC\#B}\right)}{2}$$

(3) $$\lambda_{long}=n_{e}\times \frac{\left(P_{CLC\#A}+P_{CLC\#B}\right)}{2}$$

where *P_CLC#A_* and *P_CLC#B_* are the pitch lengths of CLC#A and CLC#B, respectively. We substitute the parameters of the blue reflection spectra of Figure 4a,b into Equations (2) and (3) in order to obtain a *λ_short_* of 832.5 nm and a *λ_long_* of 915.8 nm, respectively. The obtained *λ_short_* and *λ_long_* approximately fit the short and long wavelengths of the reflection-band edge of the reflection spectra which are marked by blue in Figure 4a,b, respectively. Figure 5 shows the simulated spectrum of single-pitch CLCs with 20 turns of CLC helix while using 1D-DIMOS software, which is a reference for determining the short and long wavelengths of the reflection-band edges of the reflection spectra which are marked by blue in Figure 4a,c,e and Figure 4b,d,f, respectively. The pitch length and n_o_/n_e_ of the CLCs in Figure 5 are 560 nm and 1.5/1.65, respectively, and the short [long] wavelengths of the reflection-band edges of the reflection spectrum are 840 nm [924 nm] using Equation (2) [(3)] when *P_CLC#A_* and *P_CLC#B_* are the same [5]. Furthermore, we substitute the parameters of the reflection spectra that are marked by the blue color in Figure 4c,d [Figure 4e,f] into Equations (2) and (3) in order to obtain a *λ_short_* of 772.5 nm [712.5nm] and a *λ_long_* of 849.8 nm [783.8 nm], respectively. The calculated *λ_short_* of 772.5 nm [712.5nm] and *λ_long_* of 849.8 nm [783.8 nm] do not fit well the short and long wavelengths of the reflection-band edges of the reflection spectra which are marked by blue shown in Figure 4c,d [Figure 4e,f], respectively. Equations (2) and (3) can only be used to estimate the wavelengths of the reflection-band edges in Figure 4 when the difference in pitch length between CLC#A and CLC#B is small. Moreover, the amount of the red-shift [blue-shift] of the short [long] wavelength of the reflection-band from the reflection spectrum which is marked by blue to that of the reflection spectrum which is marked by yellow at the reflection of 0.25 progressively enlarges with the increase of the difference in pitch length between CLC#A and CLC#B, as shown in Figure 4a,c,e [Figure 4b,d,f]; referring to Figure 4a [Figure 4b] {Figure 4e [Figure 4f]}, the wavelength difference between the short [long] wavelengths of the reflection-bands of the reflection spectra that are marked by blue and yellow colors at the reflection of 0.25 is around 2.5 nm {5 nm} when the pitch length difference is 10 nm {170 nm}. Meanwhile, by substituting the parameters of the pitch lengths of CLC#A and CLC#B that are shown in Table 1a into Equation (1), Table 2 shows the corresponding *λ_cen_* of 882, 874.1, 842.6, 811.1, 779.6, 748.1, 716.6, 685.1, and 653.6 nm, which approximately fit the CRWs shown in Table 1a. These results indicate that the use of Equation (1) is nearly independent of the difference in pitch length between the CLC#A and CLC#B of BLCPFs. Furthermore, the reflection and reflection bandwidth of the other reflection bands, which are excluded within the black dashed and red dashed circles presented in Figure 3, increase with the increase of the difference between n_e_ and n_o_, and their reflection bands red-shift (blue-shift) with the increase (decrease) of n_e_ (n_o_) when n_o_ (n_e_) is fixed. Overall, when the pitch length of CLC#B is larger than half of the pitch length of CLC#A, we deduce that the reflections of reflection bands, which are unaffected by variations in LC birefringence, are CLC-like reflection bands. Conversely, the reflections of reflection bands, depending on variations in LC birefringence are 1D-PC-like reflection bands. The reflection of 1D-PC varies with the differences in thickness and refractive index between the two dielectric materials [1,2,3,4]. Accordingly, the variations in the reflection of the 1D-PC-like reflection bands in the black dashed frames B, C, D, and A-1 shown in Figure 2 and the 1D-PC-like reflection bands in Figure 3 can be qualitatively understood through the mechanism of variation of reflection bands of 1D-PCs. Accordingly, the third rule is that the reflections, bandwidths, and positions of the reflection bands of BLCPFs can be determined by n_e_ and n_o_, as well as the difference between n_e_ and n_o_; this rule is similar to that of 1D-PCs. Equations (1)–(3) are only used to elucidate CLC-like reflection bands, and the limitation of the use of Equations (2) and (3) is discussed. Moreover, Equation (1) can be used in order to determine which reflection band among a reflection spectrum is a CLC-like reflection band, and the rest are considered as 1D-PC-like reflection bands in any BLCPF.

### 3.2. Application Concept of BLCPFs in LC Displays

**Figure 6:**
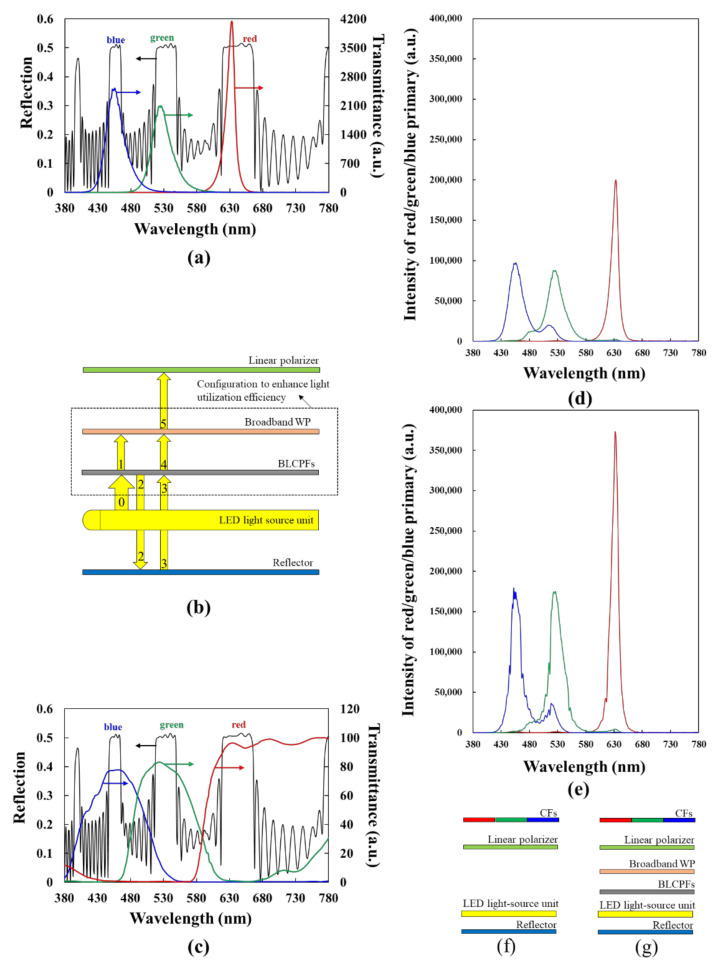
(**a**) Simulation results of the reflection spectrum (black curve) of a designed BLCPF containing three different reflection bands. The red, green, and blue curves are spectra of three emission bands of a LED light source. (**b**) Configuration of the usage of the designed BLCPFs and a broadband WP to improve the light-utilization efficiency and color gamut of LCDs/light-source unit based on (**a**). (**c**) Simulation results of the reflection spectrum (black curve) of a designed BLCPF, which is identical to that in (**a**). The red, green, and blue curves are the transmittance spectra of red, green, and blue CFs. The calculated intensities without [with] the use of the designed BLCPFs and broadband WP (*a_c_w/o_*) [(*a_c_w/_*)] of red, green, and blue primary colors as functions of wavelength (λ) curve (**d)** [(**e**)] using the configuration plotted in (**f)** [(**g**)]. (**f**) and (**b**) are the same except that the CFs and the BLCPFs with the broadband WP are added and removed in (**f**), respectively; (**g**) and (**b**) are the same, except that the CFs are added in (**g**).

The BLCPFs can be applied to various optical devices, such as RGB reflectors, color filters, and asymmetrical transmission systems, backlight enhancement films [5,7,14,26,27,28,29,30,31,32]. An application of the BLCPFs is elucidated as follows. Figure 6a shows the simulation results of the reflection spectrum of a BLCPF [black curve], for example. Figure 6a does not consider the refractive-index dispersion of LCs. The parameters for the BLCPF used in Figure 6a are as follows: (i) the pitch length of CLC#A (CLC#B) is 680 (340) nm and (ii) the n_o_ (n_e_) of the LCs is 1.45 (1.72). While using Equation (3), the reflection band covering a wavelength of 891 nm is a CLC-like one. Figure 6a presents a spectrum of a light-emitting diode (LED) light source comprising red, green, and blue emission bands [red, green, and blue curves], for example to elucidate the application of the BLCPFs [33,34]. The multiple reflection bands of the BLCPFs simultaneously include the peak-intensity wavelengths of the three emission bands of LED light source. Intrinsically, the light-utilization efficiency of the light source of LC displays (LCDs) is extremely low, primarily owing to the light absorption by linear polarizers [29,30,31,32]. Figure 6b shows the backlight unit that is embedded with the BLCPFs and a broadband wavelength plate (WP) in order to improve the light-utilization efficiency of the LED light source [29,30,31,32,35,36]. The parameters of the BLCPFs that are used in Figure 6b and those used in Figure 6a are identical. The unpolarized light (denoted by 0 in Figure 6b) from the LED light-source unit, which consists of a diffuser, edge-lit LED (whose emission bands are shown in Figure 6a), a waveguide, a prism array, and others [29,30,31,32,37,38,39,40,41], propagates toward the BLCPFs (whose reflection bands are marked by black color in Figure 6a). A reflector is placed on the backside of the light-source unit. If CLC#A and CLC#B are right-handed, then left-handed circularly polarized light (CPL) (denoted by 1 in Figure 6b) passes through the BLCPFs, and right-handed CPL (denoted by 2 in Figure 6b) is reflected. Thereafter, the reflected circularly polarized light is reflected by the reflector with depolarization in order to become unpolarized light (denoted by 3 in Figure 6b), and only the left-handed CPL passes through the BLCPFs and the right-handed CPL is reflected [32,40]. The left-handed CPL (denoted by 1 and 4 in Figure 6b) transforms into linearly polarized light (denoted by 5 in Figure 6b) after passing through a broadband WP. Eventually, the light that passes through the broadband WP passes through the linear polarizer [32,35,36]. The BLCPFs select the right polarization (left-handed CPL) to let it pass and the wrong polarization (right-handed CPL) to be reflected by repeating the process. Notably, the configuration still works if the reflector shown in Figure 6b has the function of polarization conversion [40]. The CPLs are used to elucidate the operation that is shown in Figure 6b; the actual polarization of light exiting the BLCPFs is not exactly a CPL, which will be discussed later. Most importantly, the BLCPFs serving as a CF with a broadband WP can improve the light-utilization efficiencies of LED light source for the red, green, and blue emission wavelength ranges within the reflection bands of BLCPFs, but the light-utilization efficiencies of the light wavelength outside the reflection bands cannot be effectively enhanced. Accordingly, if the light-utilization efficiency of wavelength ranges around the peak intensities of the three primary colors can be effectively enhanced, then the expansion of the color gamut of the light source is realized simultaneously.

Figure 6c shows the simulation results of the reflection spectrum of BLCPF [black curve], which is identical to that plotted in Figure 6a, and the transmittance spectra of red, green, and blue CFs [34]. The wavelength ranges of the color crosstalk of the blue (green) and green (red) CFs are partially outside the reflection bands. This finding indicates that the color gamut of LCDs can be expanded, because the wavelength ranges of some color crosstalk areas that cause color gamut reduction are not enhanced by the BLCPFs. This concept is similar to that of eliminating undesirable wavelengths in a light source in order to expand the color gamut of light sources/LCDs, which has been previously reported [27,28]. Figure 6d [6e] shows the calculated intensities without [with] the BLCPFs and a broadband WP (*a_c_w/o_*) [(*a_c_w/_*)] based on Equation (4) [(5)] of red, green, and blue primary colors as functions of wavelength (*λ*) using the configuration illustrated in Figure 6f [Figure 6g] in order to estimate the enhancement in light-utilization efficiency and color gamut. Figure 6f,b are the same, except that the CFs and the BLCPFs with a broadband WP are added and removed in Figure 6f, respectively; Figure 6g,b are the same, except that the CFs are added in Figure 6g. The details of the following two equations are discussed below.

(4) $$a_{c\_w/o}\left(\lambda \right)=S_{LED}\left(\lambda \right)\times 0.5\times CF_{c}\left(\lambda \right)$$

(5) $$a_{c\_w/}\left(\lambda \right)=S_{LED}\left(\lambda \right)\times T\left(\lambda \right)\left[1+R\left(\lambda \right)+R^{2}\left(\lambda \right)+\cdots \right]\times CF_{c}\left(\lambda \right)\;=S_{LED}\left(\lambda \right)\times T\left(\lambda \right)\left[\frac{1}{1-R\left(\lambda \right)}\right]\times CF_{c}\left(\lambda \right)$$

**Figure 7:**
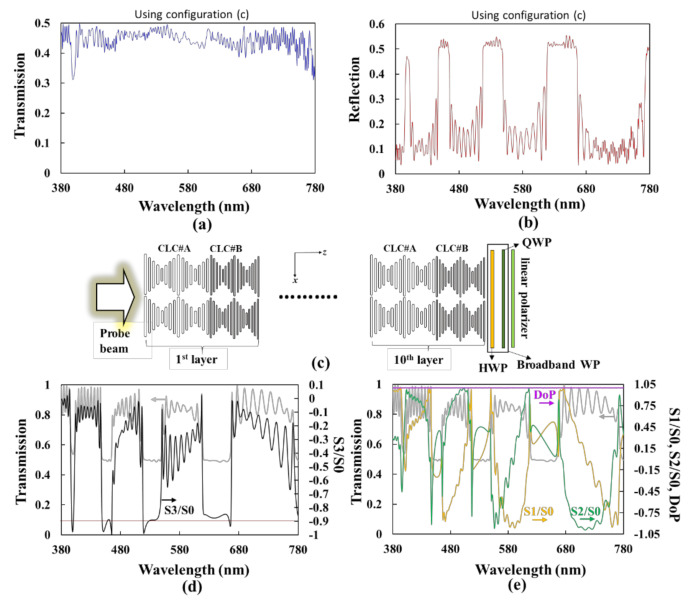
Simulation results of the (**a**) transmission and (**b**) reflection spectra, denoted as T(λ) and R(λ), respectively, in Equation (5) using the configuration of (**c**). Configuration of (**c**) is identical to that plotted in Figure 6b without the light source and reflector. (**d**) Stokes parameters of S3/S0 (black curve) as a function of the wavelength of the light passing through the BLCPFs that are shown in Figure 6b. (**e**) Stokes parameters of S1/S0 (orange curve), S2/S0 (green curve), and degree of polarization (DoP) (purple curve) as functions of the wavelength of the light passing through the BLCPFs that are shown in Figure 6b. The gray curves in (**d**,**e**) are the same and are the simulation results of the transmission spectrum of the BLCPFs as shown in Figure 6b.

Figure 7a [Figure 7b] shows the simulation result of the transmission [reflection] spectrum, which is denoted as *T(λ)* [*R(λ)*] in Equation (5), using the configuration shown in Figure 7c, which is identical to Figure 6b without the light-source unit and the reflector. The parameters of the BLCPFs that are used in Figure 7c and those used in Figure 6a,b are identical. The broadband WP presented in Figure 7c comprises a half-WP (HWP) and a quarter-WP (QWP). The angle between the slow axis of the HWP (QWP) and the ***x***-axis is 15° (75°); the transmissive axis of the polarizer is along the ***x***-axis [35,36]. The phase retardations of the HWP and QWP are 0.74π/λ and 0.37π/λ (the unit of λ is micrometer), respectively; the refractive indexes of the slow and fast axis of HWP/QWP are 1.6 and 1.5, respectively. The dispersion of the refractive-index is not considered, and the incident light is unpolarized. The subscript of *c* in Equations (4) and (5) is red, green, or blue. The *a_red_w/o_(λ)*, *a_green_w/o_(λ)*, and *a_blue_w/o_(λ)* [*a_red_w/_(λ)*, *a_green_w/_(λ)*, and *a_blue_w/_(λ)*] represent the intensities of red, green, and blue primary colors as functions of wavelength, respectively, while using the configuration of Figure 6f [6g]. The *S_LED_(λ)* is the sum of *I_red_(λ)*, *I_green_(λ)*, and *I_blue_(λ)* [*S_LED_(λ)* = *I_red_(λ*)+*I_green_(λ)* + *I_blue_(λ)*], among which *I_red_(λ)*, *I_green_(λ)*, and *I_blue_(λ)* are the intensities as functions of wavelength for the red, green, and blue LED emission bands, as shown in Figure 6a, respectively. *CF_red_(λ)*, *CF_green_(λ)*, and *CF_blue_(λ)* are the transmissive spectrum of red, green, and blue CFs, as plotted in Figure 6c, respectively. The wavelength range concerned here is from 380 nm to 780 nm (unit wavelength = 1 nm) [The unit wavelength used in all figures in this paper is 1 nm]. For simplicity, the linear polarizer is assumed to absorb 50% intensity of unpolarized light with wavelength ranging from 380 nm to 780 nm in Equations (4) and (5), and the reflector that is used in Figure 6f,g for Equations (4) and (5) is assumed to have 100% reflection ability within the wavelength range. The reflector also depolarizes the incident light into unpolarized light [40]. Here, we ignore any reflection that is caused by the light-source unit. Eventually, partial light from the light source passes through the BLCPFs, and the residual light is reflected and then depolarized and reflected in order to become unpolarized light by the reflector (refer to Figure 6b,g). This process would be infinitely repeated, and the term, $T\left(\lambda \right)\left[\frac{1}{1-R\left(\lambda \right)}\right]$, presented in Equation (5), is deduced. The calculated result of the color gamut in the International Commission on Illumination 1931 (1976) color space while using the configuration of Figure 6g increases by around 5.62% (2.22%) as compared with that on Illumination 1931 (1976) color space using the configuration of Figure 6f.

Figure 7d [Figure 7e] shows the Stokes parameters of the S3/S0 (black curve) [S1/S0 (orange curve), S2/S0 (green curve), and degree of polarization (DoP) (purple curve)] of the light passing through the BLCPFs, as shown in Figure 6b, as functions of the wavelength. The gray curves that are plotted in Figure 7d,e are the simulated transmission spectrum of the BLCPFs shown in Figure 6b. Most of the values of S3/S0 of the three bands are around −0.9, and DoP values ($\sqrt{\left(S_{1}^{2}+S_{2}^{2}+S_{3}^{2}\right)}/S_{0}$) are wavelength-independent and extremely close to 1. Moreover, the S1/S0 and S2/S0 within the three transmission bands vary with the wavelength [42].

Overall, when considering an unpolarized incident light, its transmitted light passes through the BLCPFs and its wavelength located in the transmission bands still comprises Stokes parameters of S1 and S2, which reveals that the transmitted light is not perfectly CPL light. The existence of S1 and S2 can be understood, because they are caused by a large refractive-index mismatch between LCs and air (the setting of the refractive index of the environment in the simulation in this paper is 1). A method for reducing the absolute values of S1/S0 and S2/S0 and letting the absolute value of S3/S0 approach 1 can be found in the report of Woon et al. [42].

Equation (6) [(7)] are applied in order to calculate the total intensities of the red, green, or blue primary colors that are shown in Figure 6d [Figure 6e], which is correlated with Equation (4) [(5)].

(6) $$A_{c\_w/o}=\overset{\lambda =780}{\underset{\lambda =380}{\sum }}a_{c\_w/o}\left(\lambda \right)=\overset{\lambda =780}{\underset{\lambda =380}{\sum }}\left[S_{LED}\left(\lambda \right)\times 0.5\times CF_{c}\left(\lambda \right)\right]$$

(7) $$A_{c\_w/}=\overset{\lambda =780}{\underset{\lambda =380}{\sum }}a_{c\_w/}\left(\lambda \right)=\overset{\lambda =780}{\underset{\lambda =380}{\sum }}\left[S_{LED}\left(\lambda \right)\times T\left(\lambda \right)[\frac{1}{1-R\left(\lambda \right)}]\times CF_{c}\left(\lambda \right)\right]$$

where *A_c_w/o_* [*A_c_w/_*] is defined as total red, green, or blue intensity obtained from Figure 6d [Figure 6e] (*c* = red, green, or blue). Defining the summation of three primary colors (*A_red_w/o_* + *A_green_w/o_* + *A_blue_w/o_* = *D*) and [*A_red_w/_*+ *A_green_w/_*+ *A_blue_w/_*= *E*], and $\frac{E-D}{D}$ is ~0.5998. Accordingly, light-utilization efficiency increases by ~60% when using the configuration of Figure 6b [40]. Overall, by using the configuration of Figure 6g, the simultaneous expansion of color gamut and enhancement in light-utilization efficiency of light sources/LCDs using BLCPFs become feasible. The BLCPFs, along with the broadband WP, can be applied in various types of LCDs with corresponding optimized designs. CLCs with gradient pitch lengths or stacked CLCs can also work as BLCPFs, as shown in Figure 6b. Although such kinds of CLC devices do enhance light-utilization efficiency, the color gamut cannot be effectively expanded, because the brightness of light within the wavelength range of the color crosstalk of the blue (green) and green (red) CFs causing the reduction of color gamut is also enhanced. The advantage of using CLCs with gradient pitch lengths or stacked CLCs is that they can be applied in large-size LCDs in order to enhance the light-utilization efficiency for obliquely incident light [29,30,31,32,37,38,39,40,41]. The BLCPFs in this work are valid when light travels around normally to the BLCPFs, as shown in Figure 6b. Placing a micro-prism and a lens array between the LED light-source unit and the BLCPFs shown in Figure 6b can overcome the limitation, because the obliquely incident light can be collimated to near normally enter the BLCPFs [32,43]. Moreover, a diffuser that reduces brightness must be placed on the outer side of LCDs.

Changes in LC birefringence (such as E7) are known to generally decrease with the increase of wavelength. Assuming that the n_e_/n_o_ that is used in Figure 2 is for the wavelength of 633 nm, the shifts in the positions of the reflection bands located in long wavelengths should be smaller than that located in short wavelengths if LC refractive-index dispersion is considered as compared with the original positions of the reflection bands shown in Figure 2 [24,25,32].

**Figure 8:**
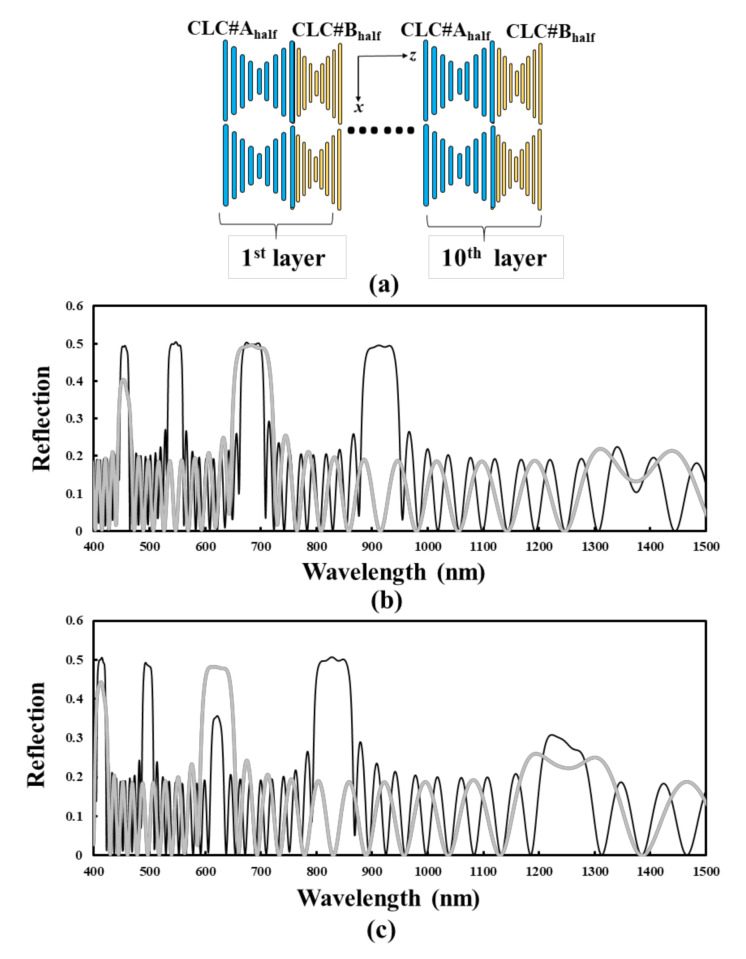
(**a**) The BLCPFs comprise of alternative layers of CLC#A_half_ and CLC#B_half_; the thickness of a single layer of CLC#A_half_ [CLC#B_half_] equals half of the single pitch length of CLC#A [CLC#B], as shown in Figure 1. The black curves are identical to the curves shown in Figure 2c with the pitch lengths of CLC#B of (**b**) 310 nm and (**c**) 230 nm. The gray curve is the simulation reflection spectrum of BLCPFs when the thickness of a single layer of CLC#A_half_ equals 280 nm, which equals half of the single CLC#A pitch length of 560 nm in Figure 2; the thicknesses of a single layer of CLC#B_half_ equal to (**b**) 155 nm and (**c**) 115 nm, which equal half of the single CLC#B pitch lengths of 310 and 230 nm in Figure 2c, respectively.

Some preliminary results are worth discussing. Figure 8a shows that the BLCPFs comprise alternative layers of CLC#A_half_ and CLC#B_half_; the thickness of a single layer of CLC#A_half_ (CLC#B_half_) equals half of the single pitch length of CLC#A (CLC#B), as shown in Figure 1. The reflection spectrum (black curve) that is plotted in Figure 8b [Figure 8c] is identical to that plotted in Figure 2c with the pitch length of CLC#A of 560 nm and the pitch length of CLC#B of 310 nm [230 nm]. The gray curve that is shown in Figure 8b [Figure 8c] is the simulated reflection spectrum of BLCPFs when the thickness of a single layer of CLC#A_half_ equals 280 nm, which equals half of the single CLC#A pitch length of 560 nm in Figure 2; the thickness of a single layer of CLC#B_half_ equals to 155 nm [115 nm], which equals half of the single CLC#B pitch length of 310 nm [230 nm] shown in Figure 2c. Figure 8b,c show that the positions of the reflection bands (gray curve) blue-shift and reflection of the right- and left-handed reflection bands (gray curve) increase when the thickness of every single layer of CLC#B_half_ decreases. Accordingly, the optical properties of the BLCPFs comprising alternative layers of CLC#A_half_ and CLC#B_half_ (Figure 8a) seem to be similar to those of BLCPFs comprising alternative layers of CLC#A and CLC#B (Figure 1). However, the number of reflection bands of the black curve is larger than that of gray ones shown in Figure 8b,c. The possible reason is that the thickness of a layer of CLC#A_half_/CLC#B_half_ of the BLCPFs of the gray curve in Figure 8b [Figure 8c] is half of that of a layer of CLC#A/CLC#B of the black curve presented in Figure 8b [Figure 8c].

### 3.3. Concept of Fabrication Method of BLCPFs

**Figure 9:**
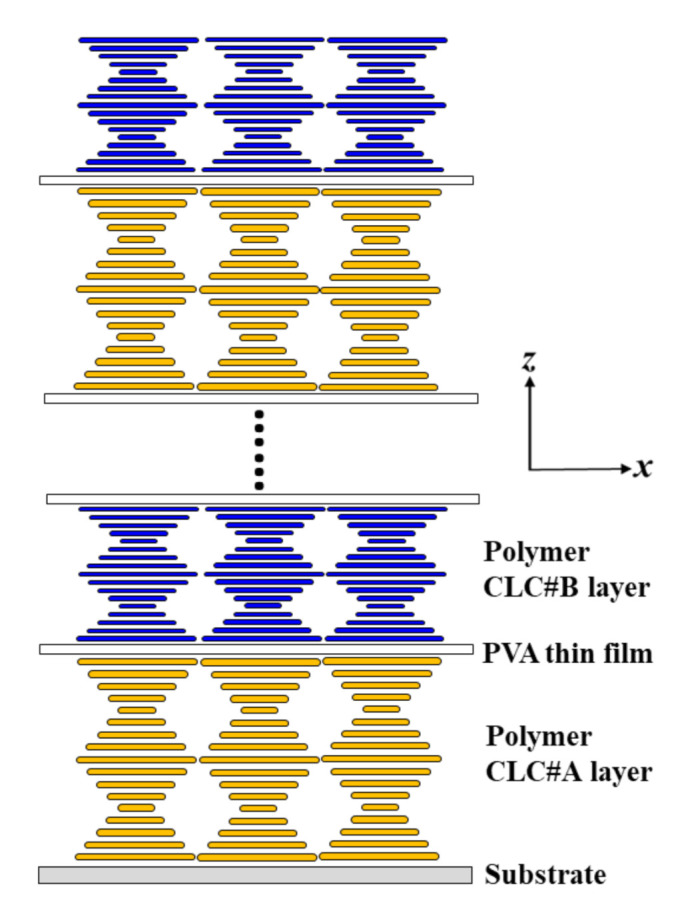
Schematic of fabrication by repeated sequentially stacking alternate polymer layer of CLC#A, poly(vinylalcohol) (PVA) thin film, polymer layer of CLC#B, and PVA thin film. PVA thin films may be replaced by PA thin films [46,47,48].

A conceptual method of fabricating the structures of BLCPFs is discussed below. The fabrication is based on the previous reports by alternately stacking different layers [7,11,44] and PVA thin film [45]. Figure 9 shows that the polymer layers of CLC#A, the PVA thin films, the polymer layers of CLC#B, and the PVA thin films are sequentially and alternately stacked upon each other in order to form BLCPFs. The fabrication of polymer layers of CLC#A is based on the report of Ohta et al. [44]. The methods of coating a PVA thin film on top of the polymer layers of CLC#A can be found in previous works [7,45]. The thickness of a PVA film should be as thin as possible to avoid its influence on the optical properties of BLCPFs. The PVA thin film and homogeneous aligned substrate generate homogeneous alignment, anchoring forces along the ***x***-axis in order to help CLCs form good planar textures with monodomain structures (or perfect planar textures) [7,26,32,44]. The polymer layer of CLC#B is then fabricated based on the report of Ohta et al. [44] on top of the PVA thin film. Another PVA thin film is then coated on top of the polymer layer of CLC#B. The thickness of each polymer layer of CLC#A or CLC#B can be precisely controlled by adjusting the solution concentration and/or the spinning speed during spin coating process [7,11,44]. Repeating the fabrications of the coating polymer layer of CLC#A, PVA thin film, polymer layer of CLC#B, and PVA thin film is possible for precisely generating the structures of the reported BLCPFs (Figure 1). Other thin films, such as photoalignment (PA) thin films [46,47,48], may replace the PVA thin films.

Notably, the optical performances/properties of CLC devices while using NLCs are usually sensitive to temperature, because the n_e_/n_o_ of NLCs generally decreases with the increase of temperature and pitch changes with a variation in temperature [32,49,50]. Accordingly, if the reported BLCPFs can be fabricated using chiral polymers and LC polymers, or polymerizable CLCs, their optical properties/performances are less sensitive to temperature [7,26,32,51,52,53,54].

## 4. Conclusions

The complete changes in reflections, reflection-wavelength ranges, and positions of spectra of BLCPFs by changing the pitch lengths and n_e_/n_o_ of CLC#A and CLC#B are investigated. The results can serve as a useful reference for the further design of BLCPFs. A way to distinguish between CLC-like and 1D-PC-like reflection bands is reported. We also report a possible method of precisely fabricating BLCPF structures. Most importantly, the rules to design BLCPFs for various applications are discussed. We also demonstrate the use of a BLCPF to enhance light-utilization efficiency and enlarge the color gamut of light source/LCDs, and more precise simulation can be made in the future. Figure 8a,b show some of the preliminary results; a full and systematic study can be further investigated. Future work can focus on developing methods of reducing the absolute value of S1/S0 and S2/S0 to approach 0 and increase the absolute value of S3/S0 to approach 1, as well as on the design of optimized reflection spectra of BLCPFs and the parameters of broadband WP in order to realize practical applications in various LCDs by considering their LC layer and other factors.

## Figures and Tables

**Table 1:** Values of FWHM, CRW, and reflection of CRW of reflection bands with reflections of CRW close to 0.5 (>0.48) in the black dashed frames (**a**) A, (**b**) B, (**c**) C, and (**d**) D plotted in Figure 2.

**(a)**										
	**Pitch Length of CLC#B (nm)**	**560**	**550**	**510**	**470**	**430**	**390**	**350**	**310**	**270**
Reflection band in the black dashed frame A	FWHM (nm)	92.7	91.9	87.5	81.8	74.3	65.3	54.9	43.6	32.2
	CRW (nm)	882.4	874.5	843.0	811.4	779.8	748.2	716.6	685.0	653.4
	Reflection of CRW	0.51	0.51	0.51	0.51	0.51	0.50	0.50	0.50	0.48
**(b)**										
		Pitch length of CLC#B (nm)				350	310	270	230	150
Reflection band in the black dashed frame B		FWHM (nm)				64.7	68.8	70.9	70.4	60.1
		CRW (nm)				958.0	915.5	872.9	830.4	745.7
		Reflection of CRW				0.49	0.50	0.50	0.51	0.50
**(c)**										
		Pitch length of CLC#B (nm)		430		390	350	310	270	230
Reflection band in the black dashed frame C		FWHM (nm)		24.6		27.3	28.4	27.8	25.0	20.2
		CRW (nm)		620.9		596.0	571.3	546.6	522.0	497.2
		Reflection of CRW		0.48		0.50	0.50	0.50	0.50	0.49
**(d)**										
				Pitch length of CLC#B (nm)				310	270	230
Reflection band in the black dashed frame D				FWHM (nm)				17.0	17.7	16.6
				CRW (nm)				455.1	434.4	413.8
				Reflection of CRW				0.49	0.50	0.50

**Table 2:** Values of *λ_cen_* of 882, 874.1, 842.6, 811.1, 779.6, 748.1, 716.6, 685.1, and 653.6 nm approximately fit the CRWs that are shown in Table 1a.

Pitch Length of CLC#B (nm)	560	550	510	470	430	390	350	310	270
CRW (nm)	882.0	874.1	842.6	811.1	779.6	748.1	716.6	685.1	653.6

## Appendix A

Figure A1 shows the simulation results plotted in Figure 2c with a wavelength range from 300 nm to 1500 nm.
